# Supplementary material for: Genomic DNA transposition induced by human PGBD5
Source: eLife. 2015 Sep 25;4:e10565. doi: 10.7554/eLife.10565 (PMC4625184; doi:10.7554/eLife.10565)
Supplement: Supplementary file 1. — DOI: http://dx.doi.org/10.7554/eLife.10565.025 [file elife10565s001.pdf]

Supplementary Table 1: Sequences of PCR primers

Name	Description	Sequence (5'-3')
PGBD5_qpcr_F	qPCR of PGBD5, forward primer	GCTTATTCTTCAGCGCATCC
PGBD5_qpcr_R	qPCR of PGBD5, reverse primer	CAGCCTCTGGGTCAGACAAT
NTerm_qPCR_F	qPCR of PGBD5 N-terminus, forward primer	AGAACATGGTGGTGCAGACA
NTerm_qPCR_R	qPCR of PGBD5 N-terminus, reverse primer	GGAGATCATGTAGCCCAGGA
TniPB_qPCR_F	qPCR of T.ni. piggyBac, forward primer	TGAGCATGGTGTACGTGTCC
TniPB_qPCR_R	qPCR of T.ni. piggyBac, reverse primer	CAGGAACATCACCTGCGACA
PB_ExPCR_F	Excision PCR assay, forward primer	GGGTTCCGCGCACATTTC
PB_ExPCR_R	Excision PCR assay, reverse primer	CAGTCATCCTCGGCAAACTCTTT
PB_qPCR_F	qPCR of transposon reporter, forward primer	GATGTCGTGTACTGGCTCCG
PB_qPCR_R	qPCR of transposon reporter	CGCGTGAAGGAGAGATGCGAG
TK1_qPCR_F	qPCR of TK1, forward primer	ATGCTGATGTCTGGGTAGGGTG
TK1_qPCR_R	qPCR of TK1, reverse primer	TGAGTCAGGAGCCAGCGTATG
Bio-linear	FLEA-PCR	[BioTEG]CATTTTGACTCACGCGGTCGT
Anchor	FLEA-PCR	GTGGCACGGACTGATCNNNNNN(N-Q)
Exponential	FLEA-PCR	GTGGCACGGACTGCA
Transposon1	FLEA-PCR	ATTGACAAGCACGCCTCACG
Transposon2	FLEA-PCR	ATGCACAGCGACGGATTCG
